# Efficacy and safety of optical coherence tomography-guided versus angiography-guided percutaneous coronary intervention: a systematic review and meta-analysis

**DOI:** 10.1097/MS9.0000000000005232

**Published:** 2026-06-10

**Authors:** Muhammad Raffey Shabbir, Allahdad Khan, Safiullah Soomro, Mujahid Ali, Maryam Tariq, Atika Shahzadi, Shafique Ahmed, Anees Ali Barkat, Muhammad Daniyal Basheer, Usha Kumari, Mohammad Ebad Ur Rehman, Huzaifa Ahmad Cheema, Asma’a Munasar Ali Alsubari, Muhammad Aslam Khan, Bilawal Nadeem, Raheel Ahmed, Adeel Ahmad

**Affiliations:** a Department of Internal Medicine, Marshfield Clinic, Marshfield, WI, USA; b Department of Medicine, Nishtar Medical University, Multan, Pakistan; c Department of Medicine, Dow University of Health Science, Karachi, Pakistan; d Department of Medicine, Rawalpindi Medical University, Rawalpindi, Pakistan; e Department of Medicine, Frontier Medical and Dental College, Abbottabad, Pakistan; f Department of Medicine, IUK ISM, Bishkek, Kyrgyzstan; g Department of Medicine, Allama Iqbal Medical College, Lahore, Pakistan; h Department of Medicine, Abbas Institute of Medical Sciences, Muzaffarabad, Pakistan; i Department of Medicine, King Edward Medical University, Lahore, Pakistan; j Department of Medicine, Sana’a University, Sana’a, Yemen; k Department of Internal Medicine, Guthrie Robert Packer Hospital, Sayre, PA, USA; l Department of Internal Medicine, Boston Medical Center, Boston, MA, USA; m Department of Cardiovascular Medicine, National Heart & Lung Institute, Imperial College London, London, UK; n Department of Cardiology, Royal Brompton Hospital, London, UK; o Department of Cardiovascular Medicine, Mayo Clinic, Rochester, MN, USA

**Keywords:** angiography, optical coherence tomography, percutaneous coronary intervention

## Abstract

**Background:**

The optimal imaging guidance for percutaneous coronary intervention (PCI) in complex coronary lesions remains a matter of debate. This meta-analysis compares the efficacy and safety of optical coherence tomography (OCT)-guided PCI versus angiography-guided PCI.

**Methods:**

Following PRISMA guidelines, we systematically searched PubMed, Embase, Cochrane, and ClinicalTrials.gov from inception to December 2024. Randomized controlled trials (RCTs) comparing OCT-guided PCI to angiography-guided PCI were included. Data analysis was performed using RevMan 5.4, with risk ratios (RR) and 95% confidence intervals (CI) calculated for dichotomous outcomes using a random-effects model. Risk of bias was assessed using the Cochrane RoB 2.0 tool.

**Results:**

Eleven RCTs involving 6432 participants were included. OCT-guided PCI showed no statistically significant reduction in all-cause mortality compared to angiography-guided PCI (RR 0.72, 95% CI 0.51–1.01, *P* = 0.06, *I*^2^ = 0%). However, OCT-guided PCI was associated with a significant reduction in cardiac death (RR 0.52, 95% CI 0.30–0.90, *P* = 0.02, *I*^2^ = 0%), major adverse cardiovascular events (MACE) (RR 0.70, 95% CI 0.55–0.89, *P* = 0.003, *I*^2^ = 0%), stent thrombosis (RR 0.53, 95% CI 0.33–0.87, *P* = 0.01, *I*^2^ = 0%), and major edge dissection (RR 0.47, 95% CI 0.34–0.65, *P* < 0.00001, *I*^2^ = 0%). Upon stratification of studies on the basis of follow-up duration, similar results were noted.

**Conclusion:**

OCT-guided PCI demonstrated significant benefits in reducing cardiac death, MACE, and procedural complications compared to angiography-guided PCI. These findings support the integration of OCT in complex PCI, although further studies are warranted to assess its impact on long-term mortality.

## Introduction

Percutaneous coronary intervention (PCI) is a key minimally invasive treatment for coronary artery disease (CAD)[1], which is one of the primary contributors to global morbidity and mortality[2]. PCI aids in reestablishing normal blood flow and alleviating symptoms associated with CAD. In 2022, approximately 315 million individuals globally were estimated to be living with CAD, highlighting its widespread prevalence[3]. PCIs are performed frequently worldwide, with approximately 600 000 procedures conducted annually across over 1600 centers in the United States[4]. Traditional angiography, the primary imaging modality used during PCI[5], provides two-dimensional lumen-level visualization, but has limitations, such as an inability to assess plaque morphology, vessel wall structure, or subtle post-stent complications^[^6–8^]^ The inability to assess the vessel wall or plaque characteristics in detail necessitates the use of complementary imaging techniques for optimal procedural outcomes. One new emerging method for guiding PCI is optical coherence tomography (OCT).

OCT is an advanced intravascular imaging technique that delivers cross-sectional images of coronary arteries with high resolution^[^9,10^]^. Unlike conventional angiography, which is limited to two-dimensional lumen visualization, OCT provides unmatched detail on vascular wall structure, stent apposition or extension, and plaque morphology^[^11–13^]^, all of which are essential for optimizing treatment strategies. OCT technology’s use in clinical practice is anticipated to grow as it develops further, offering the potential to enhance patient care by directing more specialized and tailored treatment strategies^[^14,15^]^.

Even though OCT has outperformed angiography in a number of investigations, there are conflicting data regarding its therapeutic utility in directing PCI^[^16,17^]^. However, several new RCTs have been published in recent years. A comprehensive meta-analysis synthesizing these studies is needed to resolve these discrepancies and establish whether OCT provides clear advantages over angiography in improving PCI outcomes. We conducted a systematic review and meta-analysis to evaluate the effectiveness and safety of PCI guided by OCT compared to that guided by angiography.

## Materials and methods

This meta-analysis was performed following the methodological recommendations outlined in the Cochrane Handbook for Systematic Reviews of Interventions and reported in accordance with the PRISMA guidelines^[^18,19^]^. Our protocol was registered with PROSPERO. The TITAN checklist was utilized to report the use of AI, of which there was none[20].

### Search strategy

A comprehensive literature search was carried out in MEDLINE, Embase, the Cochrane Central Register of Controlled Trials, and ClinicalTrials.gov, covering all available records from database inception through December 2024. To ensure completeness, the reference lists of eligible studies and related systematic reviews were also screened. Search terms included a combination of Medical Subject Headings (MeSH) and keywords related to optical coherence tomography, percutaneous coronary intervention, and coronary angiography.

### Eligibility criteria

We included randomized controlled trials (RCTs) that directly compared OCT-guided versus angiography-guided PCI. Studies were excluded if they were non-randomized, observational in nature, case reports, reviews, or animal studies.

### Study selection and data extraction

All search results were imported into Rayyan, where duplicate records were removed. Two reviewers (M.R.S. and M.A.) independently screened titles and abstracts to identify potentially eligible studies. Full texts of shortlisted articles were then assessed based on predefined inclusion and exclusion criteria. Discrepancies were resolved through consultation with a third reviewer (M.E.U.R.).

For all included studies, data were extracted into a structured Excel spreadsheet. Extracted variables included study characteristics (author, publication year, study location, sample size), baseline demographics, comorbidities (hypertension, diabetes, smoking, prior MI), stent type, definitions of major adverse cardiovascular events (MACE), follow-up duration, and clinical outcomes.

HIGHLIGHTS

- This meta-analysis included 11 RCTs and 6432 patients undergoing percutaneous coronary intervention (PCI).
- Optical coherence tomography (OCT) was comparable to angiography in terms of all-cause mortality.
- OCT-guided PCI was superior in terms of cardiac death, stent thrombosis, major edge dissection, and major adverse cardiovascular events.

### Outcomes

The primary outcome was all-cause mortality. Secondary outcomes included cardiac death, myocardial infarction (MI), target lesion revascularization (TLR), MACE, target vessel revascularization (TVR), stent thrombosis, stent malposition, major edge dissection, contrast-induced nephropathy, stroke, and bleeding events. TLR was defined as repeat revascularization of the target lesion after the initial PCI. TVR was defined as repeat revascularization of the target vessel after the initial PCI.

### Risk of bias assessment

The risk of bias was evaluated for the included studies using the revised Cochrane Risk of Bias tool for randomized trials (RoB 2.0)[21]. Two independent authors assessed the risk of bias for each included study, with high, low, or some concerns of bias being reported (M.T. and S.S.). Any discrepancies were resolved by a third investigator (M.E.U.R.).

### Data synthesis

All statistical analyses were undertaken using Review Manager (RevMan, version 5.4.1) and R. The Mantel-Haenszel method was used to analyze dichotomous outcomes. Risk ratios (RRs) and corresponding 95% confidence intervals (CIs) were extracted. The random-effects model was used to conduct the meta-analysis. Pooled estimates were presented as forest plots, and to assess statistical heterogeneity, the Higgins I^2^ statistic was calculated. Leave-one-out sensitivity analyses were undertaken. Subgroup analyses were performed to stratify studies based on follow-up (median follow-up ≤12 months versus median follow-up >12 months) and patient population [acute coronary syndrome (ACS) only versus stable or mixed disease]. Publication bias was assessed using Begg's and Egger’s tests for the primary outcome.

## Results

### Search results

**Figure 1.**
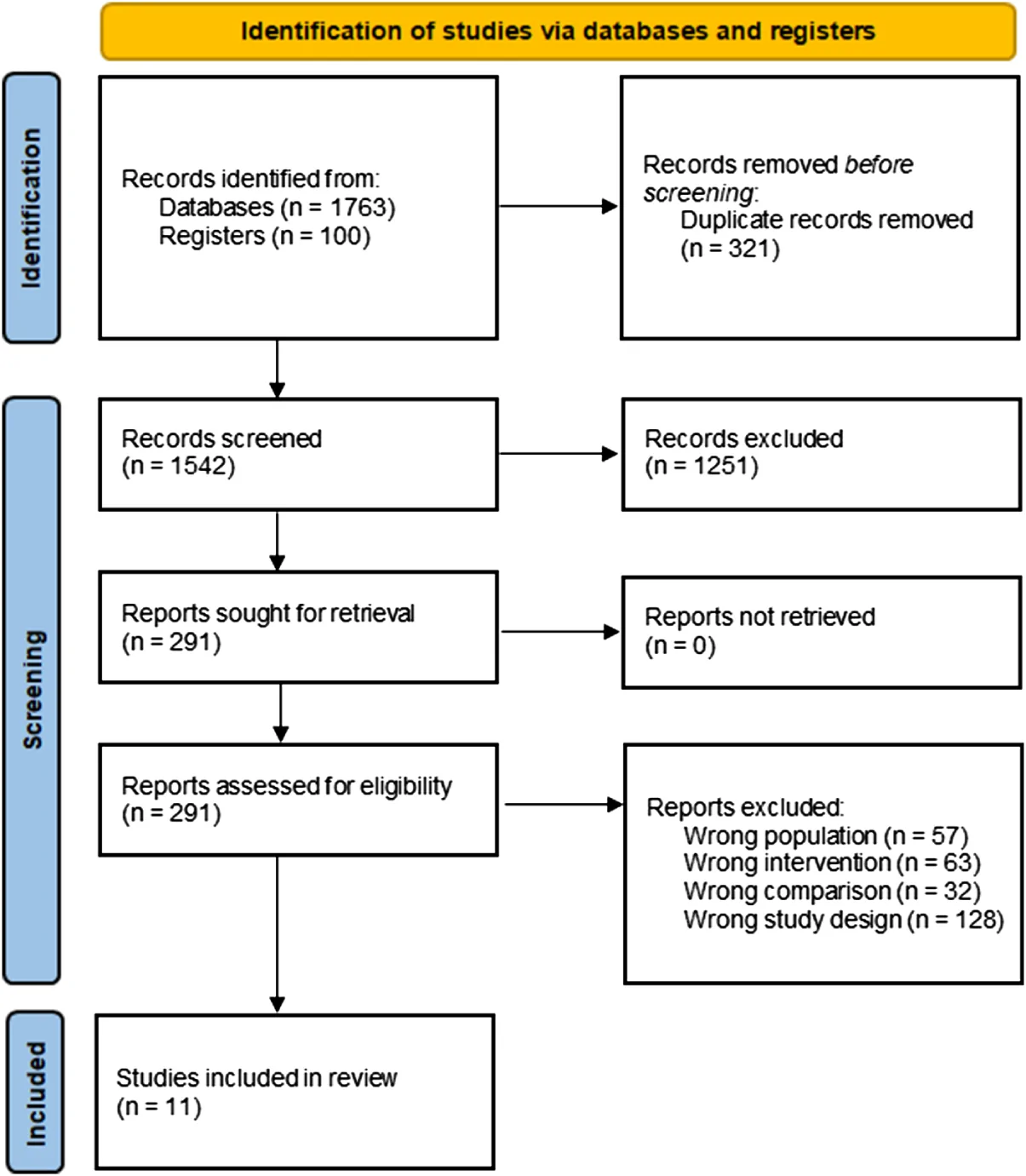
PRISMA flowchart.

The search strategy identified 1863 records; 321 duplicates were removed. 1251 studies were excluded after screening of titles and abstracts, and 280 studies were removed after full-text screening. Finally, 11 studies were included in this meta-analysis. The study selection process is depicted in Figure 1.

### Study characteristics

Using the eligibility criteria identified for this meta-analysis, 11 studies were included^[^16,22–31^]^ There were a total of 6432 patients receiving PCI: 3217 patients within the OCT-guided group and 3215 patients within the angiography-guided group. The years of publication of the included studies varied from 2015 to 2024. Details regarding study characteristics are presented in Table 1.

**Table 1:** Characteristics of included studies.

Study ID	Country	Sample size	Mean age, in years (SD)^a^	Male (%)	Inclusion criteria	HTN (%)	Diabetes (%)	Smoking (%)	Previous MI (%)	Stent type	MACE definition	Median follow-up (IQR)
Ali 2016	Eight countries	304 (158 vs. 146)	66 (59–72) vs. 67 (56–74)^b^	71	Native coronary artery with diameter 2.25–3.50 mm and length <40 mm	77	31	21	22	Everolimus-eluting, Zotarolimus-eluting, Sirolimus-eluting, Biolimus-eluting, and others	Angiographic dissection, perforation, thrombus, or acute closure of a target vessel	30 days
Ali 2023	Eighteen countries	2487 (1233 vs. 1254	65.5 (10.5) vs. 65.7 (10.3)	77	Adults ≥18 undergoing PCI with evidence of myocardial ischemia and either diabetes (on medication) or high-risk coronary lesions (recent MI culprit, long/multiple lesions >28 mm stent, complex bifurcation requiring 2 stents, severe calcification, CTO, or diffuse/multifocal ISR)	73	42	20	22	XIENCE drug-eluting stent	TVF, TVMI, TVR	729 (709–742) days
Antonsen 2015	Denmark	100 (50 vs. 50)	61.8 (9.4) vs. 62.6 (11.0)	70	NSTEMI	56	13	41	2	Biolimus-eluting stent	Subacute stent thrombosis, cardiac death	180 days
Chamie 2021	Brazil	100 (51 vs. 49)	59.92 (8.92) vs. 58.59 (10.20)	46	SA, UA, NSTEMI or STEMI	56	26	21	NR	Resolute, Xience, Promus, Biomatrix	Cardiac death, nonfatal MI, and target lesion revascularization	365 days
Holm 2023	Europe	1201 (600 vs. 601)	66.3 (10.2)	79	SA, UA or NSTEMI	72	17	Active: 14; former: 36	29	Xience everolimus-eluting stent	Cardiac cause, target lesion MI, ischemia driven target-lesion revascularization	730 days
Hong 2024	South Korea	1604 (803 vs. 801)	64 (57–70)^b^	80	Complex lesions	57	32	19	NR	Everolimus-eluting sent	Cardiac death, MI, stent thrombosis, TVR	365 (365–365) days
Kala 2018	Czech, USA	201(105 vs. 96)	57(46–70) vs. 59 (47–72)^b^	84	STEMI	50	21	61	3	Biolimus A9 or Everolimus-eluting stent	Death, myocardial infarction, target lesion revascularization	270(270–270) days
Kim 2015	South Korea	101 (50 vs. 51)	58.8 vs. 61.6	75	Age ≥20 years with significant de novo coronary lesion(s) (≥70% stenosis) in a native artery (reference diameter 2.5–4.0 mm) suitable for single-stent coverage	52	32	31	11	Zotarolimus-eluting Stent	Cardiovascular death, non-fatal MI, stent thrombosis	180 days
Meneveau 2016	France	240 (120 vs. 120)	60.8 (11.5) vs. 60.2 (11.3)	78	UA, NSTEMI	49	19	41	NR	NR	All-cause mortality, MI, stent thrombosis, TVR	180 days
Schneider 2021	Germany	56 (28 vs. 28)	70 (62–78)	72.6	Stable CAD	83.3	23.8	73.8	NR	Third-generation drug-eluting stents (DES)	Longitudinal geographic mismatch (LGM), stent edge dissections	NR
Ueki 2020	Four European countries	38 (19 vs. 19)	63.3 (12.7) vs. 62.9 (9.1)	79	Adults ≥18 with stable angina or ACS and de novo coronary lesions with reference vessel diameter 2.5–3.8 mm	47.3	21	34.2	15.7	Bioresorbable vascular scaffolds	Target lesion revascularization	360 days (IQR NR)

SA, stable angina; UA: unstable angina, STEMI, ST elevation myocardial infarction; NSTEMI, non-ST elevation myocardial infarction.

^a^ Mean age (SD) of OCT-guided PCI vs. mean age (SD) of angiography-guided PCI

^b^ Median age (IQR).

### Risk of bias in included studies

Seven studies were found to be at low risk, and two studies were found to be at high risk due to bias in randomization and in deviations from the intended intervention. Two studies were considered to have some concern of bias arising from deviations from intended interventions. This risk of bias in the included studies is showcased in Supplemental Digital Content Figure 1, available at: http://links.lww.com/MS9/B248.

### Meta-analysis of primary outcome: all-cause mortality

**Figure 2.**
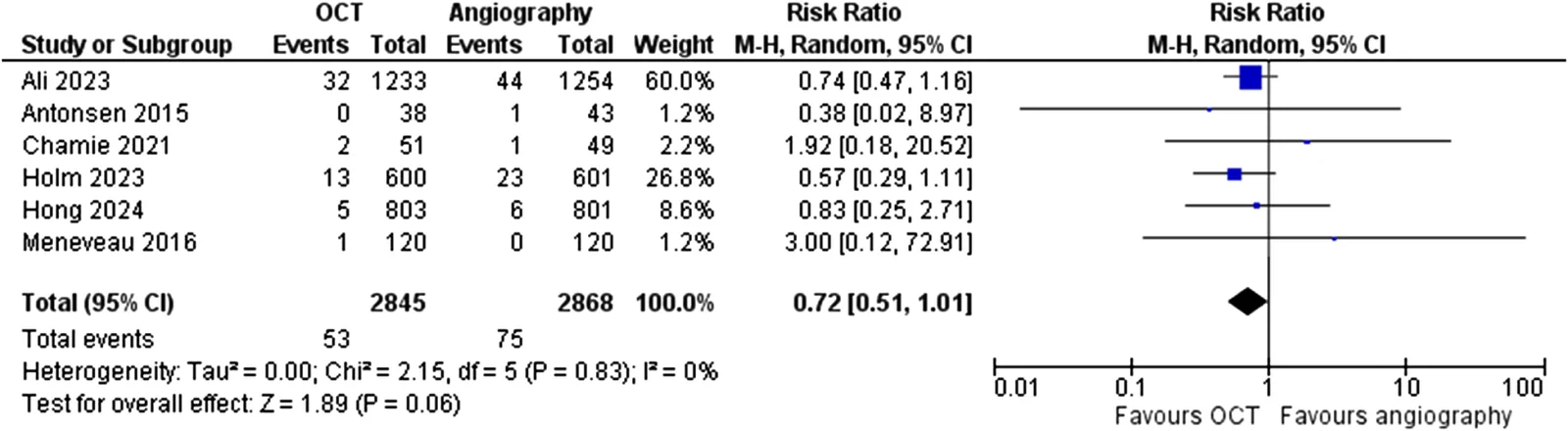
Forest plot of all cause mortality.

Six studies, involving a total of 5713 patients, were analyzed for all-cause mortality. The results indicated that OCT did not lead to a statistically significant reduction in all-cause mortality compared to the angiography group (RR 0.72, 95% CI 0.51–1.01, *P* = 0.06, *I*^2^ = 0%) (Figure 2). Leave-one-out analysis demonstrated similar results to the primary analysis. Exclusion of studies at high risk of bias yielded an RR of 0.71. Assessment of publication bias using Egger’s regression test (*P* = 0.498) and Begg’s rank correlation test (*P* = 0.272) indicated no significant evidence of asymmetry (Supplemental Digital Content Figure 2, available at: http://links.lww.com/MS9/B248).

### Meta-analysis of secondary outcomes

#### Cardiac death

Five studies, encompassing a total of 5477 patients, examined the incidence of cardiac death. The analysis demonstrated that OCT significantly reduced the risk of cardiac death compared to the angiography group (RR 0.52, 95% CI 0.30–0.90, *P* = 0.02, *I*^2^ = 0%) (Supplemental Digital Content Figure 3, available at: http://links.lww.com/MS9/B248). Leave-one-out analysis demonstrated similar results to the primary analysis.

#### MACE

Six studies, involving a total of 3405 patients, assessed this outcome. The analysis showed that OCT significantly reduced the incidence of MACE compared to the angiography group (RR 0.70, 95% CI 0.55–0.89, *P* = 0.003, *I*^2^ = 0%) (Supplemental Digital Content Figure 4, available at: http://links.lww.com/MS9/B248). Leave-one-out analysis demonstrated similar results to the primary analysis.

#### Myocardial infarction

Seven studies, comprising a total of 6128 patients, reported this outcome. The analysis indicated no statistically significant difference in the incidence of MI between the OCT and angiography groups (RR 0.82, 95% CI 0.66–1.02, *P* = 0.07, *I*^2^ = 0%) (Supplemental Digital Content Figure 5, available at: http://links.lww.com/MS9/B248). Leave-one-out analysis demonstrated similar results to the primary analysis.

#### Target lesion revascularization

Eight studies, involving a total of 6043 patients, reported on this outcome. The analysis of TLR showed no significant difference between the OCT and angiography groups (RR 0.76, 95% CI 0.48–1.20, *P* = 0.23, *I*^2^ = 33%) (Supplemental Digital Content Figure 6, available at: http://links.lww.com/MS9/B248). Leave-one-out analysis demonstrated similar results to the primary analysis.

#### Target vessel revascularization

Five studies, comprising a total of 5573 patients, evaluated this outcome. The analysis of TVR revealed no significant difference between the OCT and angiography groups (RR 0.70, 95% CI 0.43–1.16, *P* = 0.16, *I*^2^ = 53%) (Supplemental Digital Content Figure 7, available at: http://links.lww.com/MS9/B248). Leave-one-out analysis demonstrated similar results to the primary analysis.

#### Major edge dissection

Three studies, including a total of 2644 patients, assessed the incidence of major edge dissection. The analysis revealed a statistically significant reduction in major edge dissection in the OCT group compared to the angiography group (RR 0.47, 95% CI 0.34–0.65, *P* < 0.00001, *I*^2^ = 0%) (Supplemental Digital Content Figure 8, available at: http://links.lww.com/MS9/B248). Leave-one-out analysis demonstrated similar results to the primary analysis.

#### Stent malposition

Four studies involving a total of 3010 patients reported this outcome. The analysis demonstrated that OCT significantly reduced the incidence of stent malposition compared to the angiography group (RR 0.79, 95% CI 0.71–0.88, *P* < 0.0001, *I*^2^ = 18%) (Supplemental Digital Content Figure 9, available at: http://links.lww.com/MS9/B248). Leave-one-out analysis demonstrated similar results to the primary analysis.

#### Stent thrombosis

Seven studies, with a total of 5990 patients, reported this outcome. The analysis revealed a statistically significant reduction in the incidence of stent thrombosis in the OCT group compared to the angiography group (RR 0.53, 95% CI 0.33–0.87, *P* = 0.01, *I*^2^ = 0%) (Supplemental Digital Content Figure 10, available at: http://links.lww.com/MS9/B248). Leave-one-out analysis demonstrated similar results to the primary analysis.

### Subgroup analysis stratifying studies by follow-up

Subgroup analysis based on follow-up duration showed no significant subgroup differences for any outcome. For all-cause mortality, results were consistent irrespective of follow-up duration, with RR 1.06 (95% CI 0.11–10.01) at ≤12 months and RR 0.71 (95% CI 0.50–1.01) at >12 months (*P*_interaction_ = 0.73). For cardiac death, OCT showed RR 0.61 (95% CI 0.03–14.43) at ≤12 months and RR 0.53 (95% CI 0.30–0.92) at >12 months. The subgroup difference was not significant (*P*_interaction_ = 0.73). For MACE, the ≤12-month subgroup showed RR 0.95 (95% CI 0.24–3.87), while the >12-month subgroup showed RR 0.69 (95% CI 0.54–0.88). The subgroup difference remained insignificant (*P*_interaction_ = 0.73). Major edge dissection showed reductions at both follow-up durations, with RR 0.35 (95% CI 0.20–0.60) at ≤12 months and RR 0.56 (95% CI 0.37–0.86) at >12 months (*P*_interaction_ = 0.73). For MI, RRs were 1.18 (95% CI 0.32–4.35) at ≤12 months and 0.81 (95% CI 0.65–1.00) at >12 months (*P*_interaction_ = 0.73) (Supplemental Digital Content Figure 11, available at: http://links.lww.com/MS9/B248). For stent malposition, results were similar across subgroups, with RR 0.78 (95% CI 0.52–1.19) at ≤12 months and RR 0.75 (95% CI 0.70–0.79) at >12 months (*P*_interaction_ = 0.73). For stent thrombosis, RRs were 0.76 (95% CI 0.17–3.52) at ≤12 months and 0.51 (95% CI 0.31–0.86) at >12 months. The subgroup difference remained insignificant (*P*_interaction_ = 0.63). For TLR, RRs were 1.17 (95% CI 0.36–3.81) at ≤12 months and 0.67 (95% CI 0.35–1.28) at >12 months (*P*_interaction_ = 0.41). For TVR, the ≤12-month subgroup showed RR 1.48 (95% CI 0.25–8.82), and the >12-month subgroup showed RR 0.65 (95% CI 0.37–1.17), with no significant subgroup difference (*P*_interaction_ = 0.40) (Supplemental Digital Content Figure 12, available at: http://links.lww.com/MS9/B248).

### Subgroup analysis stratifying studies by patient

In subgroup analyses comparing ACS-only populations versus stable or mixed cohorts, there was no evidence of effect modification across outcomes. For all-cause mortality, the RR was 1.06 (95% CI 0.11–10.01) in ACS-only studies versus 0.71 (95% CI 0.50–1.01) in stable or mixed populations (*P*_interaction_ = 0.73). Similarly, for cardiac death, the RR was 0.61 (95% CI 0.03–14.43) versus 0.53 (95% CI 0.30–0.92), respectively (*P*_interaction_ = 0.93). No significant interaction was observed for MI (RR 1.00 [0.06–15.80] vs. 0.81 [0.65–1.01]; *P*_interaction_ = 0.88), MACE (0.22 [0.01–4.54] vs. 0.70 [0.55–0.89]; *P*_interaction_ = 0.46), TVR (2.00 [0.18–21.76] vs. 0.67 [0.39–1.14]; *P*_interaction_ = 0.38), stent thrombosis (0.62 [0.08–4.99] vs. 0.53 [0.32–0.87]; *P*_interaction_ = 0.88), or stent malposition (1.05 [0.60–1.85] vs. 0.74 [0.70–0.79]; *P*_interaction_ = 0.22).

## Discussion

In this meta-analysis involving 11 randomized controlled trials with a total of 6432 participants, we assessed the outcomes of PCI guided by OCT in comparison with those guided by angiography. As per our systematic analysis, OCT shows better outcomes for cardiac death, MACE, major edge dissection, stent malposition, and stent thrombosis, but no significant improvement in all-cause mortality, MI, TLR, and TVR.

Our meta-analysis demonstrates that OCT-guided PCI does not reduce all-cause mortality. These results align with those of several earlier meta-analyses. Kuno *et al* analyzed 32 eligible trials with a population of 22 684 patients while comparing imaging- or functionally guided versus conventional angiography-guided PCI. They reported no significant difference in all-cause death with low heterogeneity (*I*^2^ = 11.8%; *P* = 0.33) and inconsistency (*P* = 0.19)[32]. Another study by Safi *et al*, encompassing 20 RCTs and 11 698 study participants, claimed similar results (RR 0.81, 95% CI 0.64 to 1.02; *P* = 0.07; *I*^2^ = 0%)[33]. Amin *et al* examined 36 RCTs with a total of 17 572 patients, supporting similar findings (OR: 0.75, 95% CI 0.49 to 1.22)[34]. Yasmin *et al* analyzed 14 RCTs with a total of 8946 CAD patients and yielded comparable results (RR 0.85, 95% CI 0.63–1.15)[35]. On the contrary, Stone *et al* analyzed 22 trials with 15 964 patients and reported a reduction in mortality from all causes (RR 0 · 75, 95% CI 0.60 to 0 · 93; P*P* = 0.0091)[36]. A meta-analysis by Larissa *et al*, including 15 RCTs and 14 109 patients, also reported improved all-cause mortality with OCT guidance (RR 0.71, 95% CI 0.55–0.91; *P* < 0.01)[37]. Attar *et al* reviewed 21 studies, including 10 randomized controlled trials and 11 observational studies, with a total of 11 163 participants, and found lower odds of overall mortality in PCI guided by OCT (OR 0.56, 95% CI 0.48–0.67)[38]. Collectively, although several studies do report improved mortality with OCT guidance, most of the previously available evidence reinforces our findings, which observed no significant difference in all-cause mortality when comparing OCT-guided PCI with angiography-guided PCI. Compared with conventional angiography, OCT focuses on enhancing procedural outcomes and precision, which may not necessarily lead to improved long-term survival. Both interventions have limited influence on the progression, pathophysiology, and underlying risk factors for CAD.

Additionally, our analysis demonstrates that OCT considerably reduces the incidence of cardiac death and MACE in comparison with angiography-guided PCI. Kuno *et al* also reported similar results (MACE: RR 0.72, 95% CI 0.62–0.82; cardiac death: RR 0.56, 95% CI 0.42–0.75)[32]. Likewise, Safi *et al* showed a reduced risk of cardiac-related mortality (RR 0.53, 95% CI 0.39–0.72, *P* <0.001)[33]. Results from our analysis are complemented by studies done by Ashraf *et al*, Amin *et al*, Yasmin *et al*, Niu *et al*, Stone *et al*, and Larissa *et al* that demonstrated significantly reduced incidence of cardiac death and MACE^[^34–37,39,40^]^. OCT provides detailed imaging to ensure full lesion coverage, quantification of specific lesions, and vessel characteristics, ensuring more procedural precision, leading to a reduction of suboptimal outcomes that could trigger adverse events or cardiac death.

Moreover, our analysis indicates no statistically significant difference between the OCT-guided group when compared to the angiographic-guided PCI for TVR and TLR. The results were surprisingly contradictory to the already published meta-analyses by Safi *et al*, Kuno *et al*, Yasmin *et al*, Sergio *et al*, Niu *et al*, Stone *et al*, and Larissa *et al* ^[^32,33,35–37,39,41^]^ Even though OCT enhances the precision and deployment of stent placement, it does not affect the systematic and patient-oriented factors in preventing the progression of CAD, which contributes to revascularization needs.

OCT is increasingly gaining appreciation for its role in decoding the pathology of ACS and in directing its intervention. OCT can easily confirm the rupture and erosion of plaques, as well as nodular calcification, which can cause sudden coronary deaths by forming clots in ACS. Research in ACS patients has demonstrated that the culprit lesion plaque exhibited greater pan-coronary plaque vulnerability and was linked to poorer clinical outcomes compared to plaque erosion^[^42,43^]^. This would, therefore, make OCT a more accurate method for assessing the pathology of ACS, with significant prognostic and diagnostic advantages that ultimately might improve clinical outcomes[44]. In addition, under the guidance of OCT, PCI effectively reduces calcium thickness to a marked extent following stent expansion in cases of coronary calcification[45]. The usage rate of OCT in managing primary PCI in ACS is markedly lower than intravenous ultrasound, despite the gradual increase in its clinical application.

Even with its evident advantages, intravascular imaging of coronary arteries is still rarely used in practice. Current survey results identified the primary reasons for resisting the utilization of intravascular imaging: high expenses, lack of clinical benefit, and uncertainty regarding interpretation abilities and the optimal response to stenting. In reality, with intravascular imaging guidance, the cost will be more efficient over time, reducing the need for future interventional procedures and hospital readmissions[46].

Several limitations of our study should be mentioned. First, baseline characteristics varied considerably across the included studies. For example, the prevalence of hypertension ranged from 47% to 83%, diabetes from 13% to 42%, smoking from 19% to 74%, and prior MI from 2% to 29%. While patients with diabetes undergoing PCI are generally at higher risk of restenosis, secondary analyses from large-scale RCTs demonstrated no difference in the efficacy of OCT-guided PCI between diabetics and non-diabetics[47]. The impact of other patient characteristics, such as hypertension, prior MI, and smoking, on the efficacy of OCT-guided PCI remains to be elucidated. Second, while the protocols for PCI optimization using OCT may differ among trials, there is no consensus on the criteria for achieving optimal stent expansion. In addition, optimal stenting criteria were met with varying degrees of PCI procedures across the trials. Third, some of the included studies were at high risk of bias due to issues in randomization and deviation from intended interventions, which can introduce significant selection bias, leading to overestimation or underestimation of treatment effects. Fourth, although Egger’s and Begg’s tests were performed, the small number of included studies (<10) limits the reliability of these assessments for detecting publication bias.

Finally, there is likely clinical heterogeneity between stable CAD and ACS populations, which may have introduced bias into the pooled estimates despite our subgroup analyses. Although interaction testing did not demonstrate significant effect modification, the presence of mixed-population studies and the limited number of studies within each subgroup reduce statistical power and may obscure true differences between ACS and stable cohorts.

## Conclusion

Our analysis exhibited beneficial outcomes with OCT-guided PCI when compared with conventional angiography in CAD patients for stent implantation, MACE, cardiac death, stent thrombosis, and stent malpositioning. However, the efficacy of OCT did not exert beneficial effects on all-cause mortality, MI, TLR, and TVR. Supplemental and additional RCTs encompassing larger and more diverse population sizes and longer follow-up intervals are warranted for further delineating the beneficial effects of OCT in guiding PCI.

## Data Availability

The datasets will be provided by the corresponding author upon reasonable request.

## Research registration unique identification number (UIN)

Prospero: CRD42025639387.
